# Antenatal Physical Activity Interventions and Pregnancy Outcomes: A Systematic Review and Meta‐Analysis With a Focus on Trial Quality

**DOI:** 10.1111/1471-0528.18084

**Published:** 2025-02-03

**Authors:** Amanda J. Poprzeczny, Andrea R. Deussen, Megan Mitchell, Laura Slade, Jennie Louise, Jodie M. Dodd

**Affiliations:** ^1^ Department of Obstetrics and Gynaecology, The Robinson Research Institute The University of Adelaide Adelaide South Australia Australia; ^2^ Women's and Babies Division, Department of Obstetrics and Gynaecology The Women's and Children's Hospital Adelaide South Australia Australia; ^3^ Women's and Children's Hospital Research Centre Adelaide South Australia Australia; ^4^ Biostatistics Unit South Australia Health and Medical Research Institute Adelaide South Australia Australia

**Keywords:** antenatal physical activity, gestational weight gain, large for gestational age, systematic review

## Abstract

**Background:**

Guidelines recommending regular physical activity in pregnancy for improving pregnancy outcomes are informed by published meta‐analyses. Inclusion of randomised trials of poor methodological quality may bias effect estimates.

**Objectives:**

To assess the validity of these recommendations by focusing on trial quality.

**Search Strategy:**

Systematic search of PubMed, PubMed Central, Ovid Medline, Embase, Cochrane Central Register of Controlled Trials, and CINAHL from inception to 14 December 2023.

**Selection Criteria:**

Randomised trials evaluating an antenatal physical activity intervention alone, compared with no such intervention.

**Data Collection and Analysis:**

Trial quality was assessed using the Cochrane Risk of Bias tool. Independent of this, studies were grouped based on degree of deviation from the intention to treat principle. Sequential meta‐analysis was performed in which greater degrees of potential bias were allowed. Between intervention group comparisons used, relative risks or mean differences with 95% confidence intervals for dichotomous outcomes and continuous outcomes, respectively.

**Main Results:**

Overall, the quality of trial reporting was low. Only 5 trials (12.5%) were performed and analysed in keeping with the intention to treat principle. When considering only those trials performed rigorously, there was no evidence that antenatal physical activity improves pregnancy outcomes or limits gestational weight gain (WMD −0.60 kg; 95% CI −2.17, 0.98 WMD −0.60 kg; 95% CI −2.17, 0.98).

**Conclusions:**

When considering only trials at no/negligible risk of bias, antenatal physical activity interventions were not associated with improved pregnancy outcomes. Most trials were not methodologically rigorous. Incorporation of such meta‐analyses into pregnancy care guidelines may result in inaccurate recommendations.

## 1 Introduction

Multiple systematic reviews and meta‐analyses have considered the question of whether antenatal physical activity, compared with no such intervention, can improve pregnancy outcomes [1, 2, 3, 4, 5, 6, 7]. Reported benefits of a physical activity intervention include a reduction in gestational weight gain and the risk of adverse pregnancy outcomes such as preeclampsia and gestational diabetes mellitus (GDM). Such reviews have informed national and international pregnancy care guidelines, with recommendations that pregnant women engage in regular physical activity in pregnancy [8, 9, 10].

The principle of systematic reviews and meta‐analyses is to synthesise the available evidence, generating an aggregate effect estimate from multiple studies [11]. As part of this process, assessment of individual trial quality is generally undertaken, commonly utilising the Cochrane Risk of Bias tool 2.0 [12], to assess risk of bias across multiple domains, including the impact of missing outcome data. A central tenet of a rigorous randomised trial is the inclusion of outcome data from all participants recruited and randomised in an intention‐to‐treat (ITT) analysis. This is in contrast to a per‐protocol analysis, where only outcomes from those participants who followed the trial protocol are included [13].

Failure to undertake an ITT analysis introduces bias reflecting not only the extent of missing outcomes but also the degree of “missingness”, which can be related to the nature of the intervention. This is of particular relevance when considering physical activity interventions, where the underlying characteristics and clinical outcomes of those who comply are likely different from those who do not. Furthermore, exclusion of participants who are non‐compliant with an intervention limits the generalisability of the findings into clinical practice.

Our aim was to conduct a methodologically rigorous systematic review to evaluate physical activity interventions in pregnancy on clinical pregnancy outcomes and to assess the impact of including studies at increasing risk of bias on the validity of recommendations for clinical practice guidelines.

## 2 Methods: (Include Discussion on Core Outcome Sets and Patient Involvement)

This systematic review forms part of a previously reported systematic review and meta‐analysis (Dodd et al.) and was prospectively registered on PROSPERO (CRD42022324220).

### 2.1 Search Strategy

The search strategy has been previously reported and is attached as an appendix (S1). In brief, we systematically searched the electronic databases PubMed, PubMed Central, Ovid Medline, Embase, Cochrane Central Register of Controlled Trials, and Cumulative Index to Nursing and Allied Health Literature (CINAHL) from inception to 14/12/2023. Two authors (two of AP, AD, MM, LS) independently assessed each title, abstract, and full text for inclusion. Disagreements were resolved by review by a third author. Bibliographies of included studies and systematic reviews were hand‐searched to identify any additional studies.

### 2.2 Study Selection

This systematic review considered all randomised trials that evaluated the effect of an antenatal physical activity intervention alone (i.e., the intervention did not include dietary or behavioural components). Cluster‐randomised trials were included, with appropriate methodology to account for clustering, and relevant arms of multi‐armed trials were also eligible for inclusion. For cluster randomised trials, reported means and standard deviations (or frequencies for dichotomous outcomes) were considered for inclusion in the meta‐analysis if sufficient information (number of clusters, total number of participants, and an estimate of the intraclass cluster coefficient) was provided to allow for adjustment of standard errors. The online Covidence (Veritas Health Innovation, Melbourne, Australia) tool was used for study screening and data extraction.

Studies were excluded if they were not available in full in the English language, in abstract form only, performed solely among women recruited prior to planned ART/IVF, or if they recruited women with specific medical conditions (i.e., diabetes mellitus, PCOS). Studies were also excluded if they were determined not to be randomised. Trial registrations and study protocols, if available, were searched; studies resulting from the same trial registration were only included in the meta‐analysis once, using the most complete and earliest publication as the primary trial report.

### 2.3 Study Quality Assessment

Two review authors (two of AP, AD, MM, and LS) independently assessed risk of bias using the Cochrane Risk of Bias tool [14]. This tool assesses the risk of bias of included trials under the following domains: random sequence generation, allocation concealment, blinding of participants and personnel, blinding of outcome assessment, incomplete outcome data, selective reporting, and other sources of bias. Each domain was rated as low or high risk of bias where explicit statements pertaining to each specific domain could be identified in the trial report and as unclear where there was insufficient information available to make a judgement. Any discrepancies were resolved through discussion and review by a third reviewer.

### 2.4 Assessment of Study Degree of Departure From the Intention to Treat Principle

Studies were further assessed according to the degree of deviation from an ITT analysis as:

1. No or negligible potential for bias (no post‐randomisation exclusions from the analysis population, or the exclusions were considered to be of negligible impact);
2. At most minimal potential for bias (post‐randomisation exclusions occurred but were expected to have a minimal impact on the estimate of intervention effect);
3. At most moderate potential for bias (ITT status was unclear due to insufficient information on participant flow or numbers analysed, or data were not collected or used from participants who discontinued the study); and
4. Substantial potential for bias (participants were excluded for non‐compliance or if they experienced post‐randomisation events known to be related to outcomes).

Further details are provided in Table 1.

**TABLE 1:** Classification of degree of potential for bias based on departures from the intention‐to‐treat population.

Degree of potential bias	Definition
No or negligible	Those studies where there were no post‐randomisation exclusions from the analysis population or where any post‐randomisation exclusions were considered to be of negligible impact *Justification* Studies included in this group demonstrated evidence of an attempt to collect outcome data from all randomised participants (including those who discontinued the study, were subsequently found to be ineligible after randomisation, or had post‐randomisation events affecting the primary trial outcome), and all available data were included in the analysis
At most minimal	Those studies where post‐randomisation exclusions occurred but were expected to have a minimal impact on the estimate of intervention effect *Justification* While exclusion of participants found to be ineligible post‐randomisation can cause bias, this is usually because discovery of ineligibility is not random with regards to treatment. Where the number of affected participants was small in relation to the overall study, and/or the probability of discovery of ineligibility was unlikely to differ between groups, any effect on outcomes was considered minimal. This group included studies where exclusions were a very small proportion of the overall sample size and/or were unrelated to the intervention or outcomes (e.g., exclusion due to discovery of multiple gestation)
At most moderate	Those studies where intention to treat status was unclear, in addition to studies where data were not collected or used from participants who discontinued the study *Justification* Discontinuations from any study are likely not to be random in relation to the intervention or outcome, and this is particularly true of the intervention types in this systematic review. The estimate of intervention effects is therefore prone to bias if no account is taken of informative missing data. Studies were included in this group if there was insufficient information on participant flow or numbers analysed, or there was no evidence of an attempt to collect data from participants who discontinued the study
Substantial	Those studies where participants were excluded for non‐compliance *Justification* Studies included in this group had a substantial risk of potential bias. In these studies, participants were excluded, e.g., for not adhering to the intervention, for not doing enough exercise in the intervention group, and/or for doing too much exercise in the control group. In addition, some studies were also assigned to this category if participants were excluded from further data collection or analysis if they experienced post‐randomisation events known to be related to outcomes (e.g., preterm birth, GDM, or pre‐eclampsia)

### 2.5 Data Extraction

Two review authors (two of AP, AD, MM, LS) independently extracted data from each eligible study, including study details, study characteristics, and study outcomes, and checked for consistency by a third author. Trial registrations and other details (including study protocols if available) were searched. Where our outcomes of interest were reported as secondary outcomes in the included trials, available data were extracted and included in the meta‐analysis, if sufficient information was available. If any information pertaining to the study was unclear, study authors were contacted for additional details.

### 2.6 Data Analysis

Between intervention group comparisons, we used relative risks or mean differences with 95% confidence intervals (CIs) for dichotomous outcomes and continuous outcomes, respectively, with meta‐analysis using random‐effects models. We planned to use random rather than fixed‐effects models as we anticipated that statistical heterogeneity would be substantial. Statistical heterogeneity among studies was assessed by *τ* ^2^, *I* ^2^ and *Q* statistics. Small study effects were examined using funnel plots and Galbraith plots when there were sufficient studies to do so. All analyses were conducted using Stata v18.

For each outcome, a sequential meta‐analysis was performed in which studies were included with increasing potential for bias, according to the degree of deviation from the intention to treat principle, as outlined above.

## 3 Results

**FIGURE 1:**
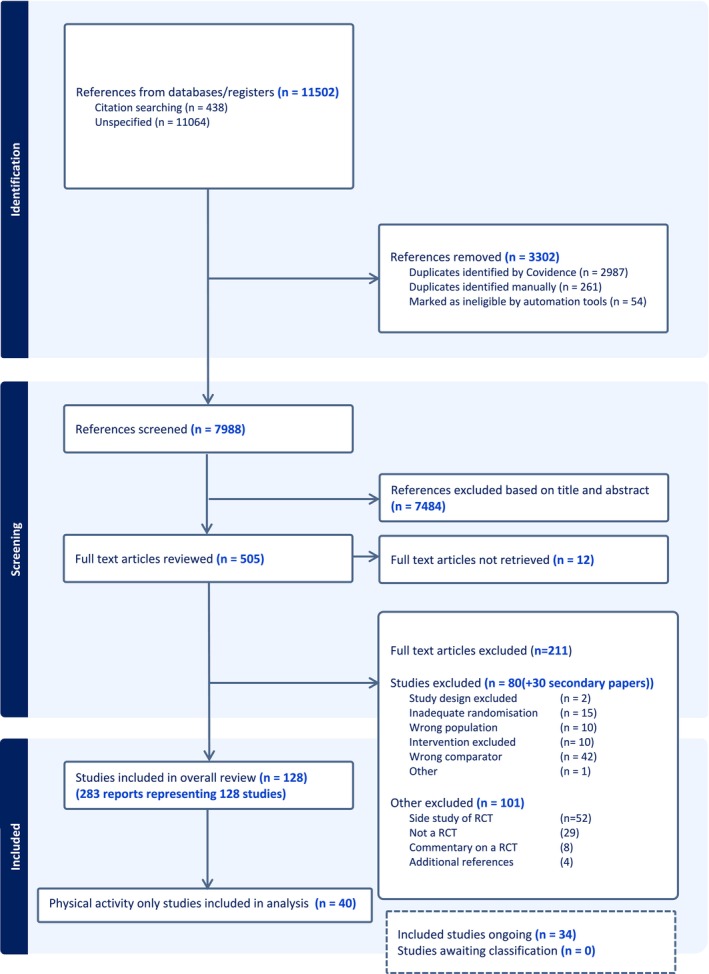
PRISMA flow chart.

The results of the primary systematic review and meta‐analysis have previously been published [15]. In brief, we identified 11 502 references from our search strategy. Of these, 3302 were removed, and 7988 titles and abstracts were screened for inclusion. Of these, 540 full‐text articles were reviewed, with 283 reports representing 128 studies included in the primary systematic review and meta‐analysis (see Figure 1). For the purposes of the current meta‐analyses, only those studies reporting outcomes from a physical activity alone intervention were included (40 studies).

### 3.1 Characteristics of the Included Studies

Included trials were conducted in Spain (18/40 studies; 45.0%), Turkey (1/40 studies; 2.5%), Argentina (1/40 studies; 2.5%), Brazil (4/40 studies; 10.0%), Denmark (1/40 studies; 2.5%), Canada (2/40 studies; 5.0%), the United States of America (4/40 studies; 10.0%), Norway (1/40 studies; 2.5%), Australia (2/40 studies; 5.0%), New Zealand (1/40 studies; 2.5%), Sweden (1/40 studies; 2.5%), Colombia (1/40 studies; 2.5%), China (2/40 studies; 5.0%) and Iran (1/40 studies; 2.5%) (see Table 2). Included trials were published between 1999 and 2021, with 32 trials (80.0%) published after the instigation of the CONSORT guidelines [56]. A total of 5 trials (12.5%) were prospectively registered prior to recruitment commencing; 23 trials (57.5%) were retrospectively registered; and 12 trials (30%) did not provide sufficient information to ascertain whether participant recruitment had commenced prior to trial registration. Only 5 trials (12.5%) reported a primary outcome that was consistent with the primary outcome as recorded in the prospective trial registration or published protocol.

**TABLE 2:** Included studies.

Primary paper	City & country	Participants randomised (*n*)	BMI range (kg/m^2^)	Prospective registration (Y/N/UN)	Intervention details	Primary outcome(s) as stated in primary paper	Pre‐specified primary outcome (Y/N)
Aguilar‐Cordero 2019 [16]	Granada, Spain	140	> 12.0^b^	N	Supervised exercise program 3 days/week for duration of pregnancy	PP depression	N
Aktan 2021 [17]	Ankara, Turkey	65^a^	Not specified	UN	Pilates 2 days/week for 8 weeks	BW, labour duration, GA, Apgar scores	N
Bacchi 2018 [18]	Buenos Aires, Argentina	140	All	N	Supervised exercise 3 days/week from 8–11 weeks to 38–39 weeks	GWG, BW	N
Baciuk 2008 [19]	Sao Paolo, Brazil	71	Not specified	UN	Water aerobics 3 days/week	Cardiovascular capacity	N
Backhausen 2017 [20]	Copenhagen, Denmark	516	< 29.0	N	Unsupervised water exercise 2 days/week after the introductory session, free access to swimming pools, weekly emails	Intensity of low back pain	Y
Barakat 2008 [21]	Madrid, Spain	160	≤ 40.0	N	Supervised group exercise 3 days/week	GA at birth	N
Barakat 2011 [22]	Madrid, Spain	80	Not specified	UN	Supervised group exercise 3 days/week	Health perception, urinary incontinence	N
Barakat 2012a [23]	Madrid, Spain	320	Not specified	UN	Supervised group exercise 3 days/week	Type of delivery	N
Barakat 2012b [24]	Madrid, Spain	100	Not specified	UN	Supervised group exercise 3 days/week	Maternal glucose tolerance, GWG, GDM	N
Barakat 2013 [25]	Madrid, Spain	510	Not specified	N	Supervised group exercise sessions 3 days/week from 10–12 to 38–39 weeks	GDM	N
Barakat 2014 [26]	Madrid, Spain	251	Not specified	N	Supervised group exercise 3 days/week	Maternal and foetal parameters	N
Barakat 2016 [27]	Madrid, Spain	840	Not specified	N	Supervised group exercise 3 days/week from 9–11 to 38–39 weeks	Proportion of women P‐HT	N
Barakat 2018a [28]	Madrid, Spain	508	Not specified	N	Supervised group exercise 3 days/week from 9–11 weeks to 38–39 weeks	Duration of labour	N
Barakat 2018b [29]	Madrid, Spain	65	Not specified	N	Supervised group exercise 3 days/week	Placental weight	N
Bisson 2015 [30]	Quebec, Canada	50	≥ 30.0	N	Supervised exercise program 1 day/week + optional additional 2 days/week from 15 to 27 weeks	Physical activity level	N
Brik 2019 [31]	Madrid, Spain	120	Not specified	N	Supervised exercise program 3 days/week for the duration of pregnancy	GWG, foetal cardiac function	N
Clapp 2000 [32]	Cleveland, USA	50	All	UN	Monitored weight‐bearing exercise sessions 3–5 days/week	BW, placental growth and volume	N
Cordero 2015 [33]	Madrid, Spain	342	Not specified	N	Supervised group exercise 3 days/week	GDM	N
Da Silva 2017 [34]	Pelotas, Brazil	639	< 35.0	Y	Supervised exercise 3 days/week from 16 to 20 weeks	Preterm birth, PE	Y
De OliveriaMelo 2012 [35]	Campina Grande, Brazil	187^a^	Not specified	Y	Moderate‐intensity supervised walking 3 days/week from 13 weeks	Uteroplacental and foetal blood flow, foetal growth, PE, P‐HT, BW, infant length	Y
Garnaes 2016 [36]	Trondheim, Norway	91	≥ 28.0^c^	N	Supervised exercise 3 days/week delivery + home‐based exercise program 1 day/week; encouraged to track GWG	GWG	Y
Guelfi 2016 [37]	Perth, Australia	172	Not specified	Y	Supervised 3 days/week 14‐week stationary cycle program at home	GDM	Y
Hopkins 2010 [38]	Auckland, New Zealand	98	All	N	Home‐based exercise up to 5 days/week to 36 weeks or delivery; fortnightly supervised exercise session	Maternal insulin sensitivity, neonatal outcomes	N
Kihlstrand 1999 [39]	Falun Sweden	258	Not specified	UN	Group water gymnastics classes 1 day/week in the 2nd half pregnancy	Intensity of low back pain, number of days on sick leave	N
Kong 2014 [40]	Ames, USA	42	≥ 25.0	UN	Unsupervised walking program; treadmill provided, following training session. Logs reviewed at study visits	Pregnancy and birth outcomes	N
Marquez‐Sterling 2000 [41]	Miami, USA	20	Not specified	UN	Supervised exercise 3 days/week for 15 weeks	Maternal physical and psychological changes	N
Nascimento 2011 [42]	Campinas, Brazil	82	≥ 26.0	UN	Supervised exercise classes weekly, home exercise program 3 days/week	GWG, ex‐GWG	N
Ong 2009 [43]	Perth, Australia	12	Obese, not defined	UN	10 weeks of home‐based supervised exercise (3 days/week) on a stationary cycle	Maternal blood glucose and insulin	N
Pelaez 2014 [44]	Madrid, Spain	169	Not specified	N	Supervised group exercise 3 days/week including pelvic floor exercises	Urinary incontinence	N
Perales 2015 [45]	Madrid, Spain	129	≥ 25.0	N	Supervised group exercise 3 days/week, including pelvic floor exercises	Maternal depression, GWG	N
Price 2012 [46]	Austin, USA	91	< 39.0	N	Supervised exercise 3 days/week, and 1 day/week unsupervised walking	Cardiorespiratory fitness	N
Robledo‐Colonia 2012 [47]	Cali, Colombia	80	Not specified	Y	Supervised group exercise sessions 3 days/week from 16 to 30 weeks for 3 months	Maternal depression	N
Rodriguez‐Diaz 2017 [48]	Quiron Campo de Gibraltar, Spain	105	Not specified	UN	Pilates classes 2 days/week from 26–28 to 34–36 weeks	Composite of maternal physical condition	N
Roldan‐Reoyo 2019 [49]	Madrid, Spain	131	Not specified	N	3 days/week exercise	FHR response recovery time	N
Rong 2021 [50]	Wuhan, China	64	Not specified	N	12‐week course of yoga classes 3 days/week	Pregnancy discomfort, maternal depression and anxiety, childbirth self‐efficacy, delivery outcomes	N
Ruiz 2013 [51]	Madrid, Spain	962	Not specified	N	Supervised exercise 3 days/week	GWG	N
Sadeghi 2018 [52]	Tehran, Iran	96^a^	Not specified	N	Training session and 8 weeks walking at home with regular contact	General health	N
Silva‐Jose 2021 [53]	Madrid, Spain	122	Not specified	N	Virtual supervised exercise program 3 days/week	Ex‐GWG	N
Skow 2021 [54]	Alberta, Canada	59	Not specified	N	Supervised aerobic exercise 3–4 times/week	Sympathetic nerve activity	N
Wang 2017 [55]	Beijing, China	300	≥ 24.0	Y	Supervised cycling 3 days/week at moderate intensity	GDM	Y

*Note: Prospective registration*: Y, yes; N, No; UN, no registration reported/unclear (period of recruitment not stated)/prior to trial registry's. *Primary outcomes* are those identified by the authors in the primary paper. *Pre‐specified primary outcome*: primary outcome(s) specified in published protocol and/or prospective trial registration.

Abbreviations: BMI, body mass index; BW, infant birthweight; CS, caesarean section; EE, energy expenditure; ex‐GWG, proportion of women exceeding GWG (IOM) guidelines; FHR, foetal heart rate; GA, gestational age; GDM, gestational diabetes mellitus (as defined by trialists); IR, insulin resistance; LGA, infant born large for gestational age (> 90th percentile for gestational age); NICU, neonatal intensive care unit; PA, physical activity; PE, preeclampsia (as defined by trialists); P‐HT, pregnancy‐induced hypertension; PP, post‐partum; PPWR, post‐partum weight retention; w‐GWG, proportion of women within GWG (IOM) guidelines.

^a^ Multi‐arm studies.

^b^ Aguilar‐Cordero 2019: women < 12 kg/m^2^ excluded, but authors note that no participants had BMI < 18.5 kg/m^2^.

^c^ Garnaes 2016; BMI inclusion changed during the study from ≥ 30.0 kg/m^2^ to ≥ 28.0 kg/m^2^.

#### 3.1.1 Participants

The number of participants randomised ranged from 12 to 962, with 23 trials (57.5%) recruiting and randomising 100 or more participants. Maternal BMI at trial entry was not specified in 25 trials (30.0%), with 3 (7.5%) recruiting women of any BMI. A total of 12 trials (30%) specified a maternal BMI cut‐off at trial entry, which ranged from ≥ 18.5 kg/m^2^ to ≤ 40 kg/m^2^. A single trial stated a lower BMI cut‐off of 12 kg/m^2^; however, reported that no participants had a BMI < 18.5 kg/m^2^ (Table 2).

#### 3.1.2 Interventions

The intensity of the physical activity intervention varied from a recommended exercise regimen, to one performed under supervision at least 3 days per week, utilising supervised group exercise sessions. The nature of physical activity varied, including yoga or Pilates classes, cycling sessions, water‐based gymnastics, or walking (see Table 2).

### 3.2 Assessment of Study Quality and Adherence to the Intention to Treat Principle

#### 3.2.1 Study Quality (Risk of Bias Assessment)

A total of 23 trials (57.5%) explicitly reported use of a robust random sequence generation, while insufficient information prevented assessment in a further 17 trials (42.5%). A total of 24 trials (60.0%) did not provide sufficient information to assess allocation concealment. None of the included trials were able to blind participants and personnel to allocation group, with 10 (25.0%) providing sufficient information about blinding of outcome assessors. Post‐randomisation exclusions or losses to follow‐up were more than 20% of the recruited cohort in 26 trials (65.0%). A total of 22 trials (55%) did not provide sufficient information to assess whether selective outcome reporting had occurred, with an additional 7 trials (17.5%) at high risk of bias due to significant differences between trial registrations or published protocols and publications.

### 3.3 Assessment of Trial Degree of Departure From the Intention to Treat Principle

Five trials (12.5%) utilised an ITT analysis, with a further 3 trials (7.5%) having minimal deviations. A total of 18 trials (45.0%) had moderate deviations from the ITT analysis, while 14 trials (35.0%) excluded non‐compliant participants from analysis and therefore were not considered ITT.

### 3.4 Outcomes

#### 3.4.1 Meta‐Analysis 1: Birthweight

A total of 33 trials (82.5%) reported infant birthweight, with 31 (77.5%) contributing data to the meta‐analysis. Four trials (10%) were considered to have a negligible potential for bias, the meta‐analysis identifying no significant effect from the intervention on infant birthweight (4 trials; 247 participants; WMD 20.97 g; 95% CI −85.45, 127.40; 95% prediction interval −212.66, 254.60; *I* ^2^ = 0.00; *τ* ^2^ = 0.00) (Table 3). Sequential inclusion of trials at increasing potential for bias resulted in shifting of the estimated effect away from the null while simultaneously introducing greater heterogeneity (Figure S2). When incorporating all trials, including those with significant potential for bias, there was no significant effect of physical activity interventions on infant birthweight (31 trials; 5633 participants; WMD −26.17 g; 95% CI −56.73, 4.39; 95% prediction interval −118.42, 66.08; *I* ^2^ = 26.87; *τ* ^2^ = 1791.49) (Table 3).

**TABLE 3:** Effect of intervention on infant and maternal outcomes.

Risk of bias	*N* studies with data	Overall estimate	Prediction interval	*τ* ^2^	*I* ^2^
**Infant birthweight**		**WMD [g] (95% CI)**			
Negligible	4	20.97 (−85.45, 127.40)	−212.66, 254.60	0.00	0.00
+Minimal	6	23.13 (−45.64, 91.89)	−110.08, 156.34	1071.12	13.48
+Moderate	19	−46.85 (−88.62, −5.08)	−153.56, 59.86	2103.77	26.97
+Substantial (all trials)	31	−26.17 (−56.73, 4.39)	−118.42, 66.08	1791.49	26.87
**Gestational diabetes**		**Relative risk (95% CI)**			
Negligible	1	0.60 (0.16, 2.23)	N/A^a^	N/A^a^	N/A^a^
+Minimal	3	0.99 (0.73, 1.34)	0.14, 7.00	0.00	0.00
+Moderate	8	0.81 (0.61, 1.08)	0.43, 1.53	0.05	30.61
+Substantial (all trials)	13	0.77 (0.59, 1.01)	0.40, 1.49	0.07	36.44
**Preeclampsia**		**Relative risk (95% CI)**			
Negligible	0				
+Minimal	2	1.05 (0.54, 2.06)	N/A^a^	0.00	0.00
+Moderate	4	1.12 (0.65, 1.94)	0.34, 3.73	0.00	0.00
+Substantial (all trials)	6	0.89 (0.55, 1.44)	0.45, 1.76	0.00	0.00
**Caesarean birth**		**Relative risk (95% CI)**			
Negligible	4	0.74 (0.49, 1.12)	0.30, 1.82	0.00	0.00
+Minimal	5	0.86 (0.66, 1.12)	0.56, 1.32	0.00	0.00
+Moderate	15	0.79 (0.65, 0.95)	0.53, 1.17	0.02	18.41
+Substantial (all trials)	23	0.83 (0.74, 0.93)	0.73, 0.94	0.00	0.00
**Gestational weight gain**		**WMD [kg] (95% CI)**			
Negligible	2	−0.60 (−2.17, 0.98)	N/A^a^	N/A^a^	N/A^a^
+Minimal	4	−0.63 (−1.23, −0.02)	−1.95, 0.70	0.00	0.00
+Moderate	14	−1.26 (−1.76, −0.76)	−2.48, −0.04	0.25	33.48
+Substantial (all trials)	22	−1.22 (−1.55, −0.90)	−2.09, −0.35	0.15	28.29
**Infant large for gestational age**		**Relative risk (95% CI)**			
Negligible	1	1.33 (0.33, 5.33)	N/A^a^	N/A^a^	N/A^a^
+Minimal	4	1.00 (0.71, 1.41)	0.47, 2.12	0.00	0.00
+Moderate	5	0.88 (0.65, 1.18)	0.54, 1.42	0.00	0.00
+Substantial (all trials)	7	0.88 (0.67, 1.16)	0.61, 1.27	0.00	0.00

Abbreviation: WMD, weighted mean difference.

^a^ Unable to calculate due to small number of studies.

#### 3.4.2 Meta‐Analysis 2: Gestational Diabetes Mellitus

A total of 16 trials (40%) reported GDM, with data available from 13 trials (32.5%). Only 1 trial (2.5%) was considered to have no/negligible potential for bias, with no significant effect of the intervention on GDM (1 trial; 48 participants; RR 0.60; 95% CI 0.16, 2.23) (Table 3). Inclusion of trials at progressively increasing potential for bias shifted the estimate of effect further from the null, with progressively increasing heterogeneity (Figure S2). When incorporating all trials, there was no significant effect of physical activity interventions on GDM (13 trials; 3347 participants; RR 0.77; 95% CI 0.59, 1.01; 95% prediction interval 0.40, 1.49; *I* ^2^ = 36.44; *τ* ^2^ = 0.07) (Table 3).

#### 3.4.3 Meta‐Analysis 3: Preeclampsia

Overall, 7 trials (17.5%) reported and contributed data for the outcome of preeclampsia. No trials were considered to have negligible potential for bias. When considering only trials with at most minimal potential for bias, there was no significant effect of the intervention on preeclampsia (2 trials; 781 participants; RR 1.05; 95% CI 0.54, 2.06) (Table 3). The effect of sequential incorporation of trials at increasing potential for bias on the effect estimate is demonstrated in Figure S2. Inclusion of all trials demonstrated no significant effect of physical activity interventions on preeclampsia (6 trials; 1920 participants; RR 0.89; 95% CI 0.55, 1.44; 95% prediction interval 0.45, 1.76; *I* ^2^ = 0.00; *τ* ^2^ = 0.00) (Table 3).

#### 3.4.4 Meta‐Analysis 4: LSCS

Overall, 26 studies (65.0%) reported caesarean birth, with 23 (57.5%) contributing data. When considering only trials with no/negligible potential for bias, there was no significant effect on caesarean birth (4 trials; 252 participants; RR 0.74; 95% CI 0.49, 1.12; 95% prediction interval 0.30, 1.82; *I* ^2^ = 0.00; *τ* ^2^ = 0.00) (Table 3). Sequential incorporation of trials at increasing potential for bias resulted in shifting the estimate of effect further from the null (Figure S2). When incorporating all trials, there was a significant effect of the intervention on caesarean birth (23 trials; 3876 participants; RR 0.83; 95% CI 0.74, 0.93; 95% prediction interval 0.73, 0.94; *I* ^2^ = 0.00; *τ* ^2^ = 0.00) (Table 3).

#### 3.4.5 Meta‐Analysis 5: Gestational Weight Gain

A total of 28 trials (70%) reported gestational weight gain, with 22 trials (55.0%) contributing data for meta‐analysis. When considering trials at no or negligible risk of bias, there was no significant effect of the intervention on gestational weight gain (two trials; 113 participants; WMD −0.60 kg; 95% CI −2.17, 0.98) (Table 3). Incorporating data from trials at increasing potential for bias resulted in shifting of the estimate of effect further from the null, with progressively increasing heterogeneity (Figure S2). When incorporating all trials, there was a modest significant effect on gestational weight gain (22 trials; 4111 participants; WMD −1.22 kg; 95% CI −1.55, −0.90; 95% prediction interval −2.09, −0.35; *I* ^2^ = 28.29; *τ* ^2^ = 0.15) (Table 3).

#### 3.4.6 Meta‐Analysis 6: Large for Gestational Age Infant

Only 7 trials (17.5%) reported the number of infants born large for gestational age (LGA). When considering only trials with negligible potential for bias, there was no evidence of a significant effect on infant LGA (1 trial; 48 participants; RR 1.33; 95% CI 0.33, 5.33) (Table 3). Sequential incorporation of trials at increasing potential for bias resulted in shifting the estimated effect further from the null (Figure S2). When including all trials, there remained no significant effect on infant LGA (7 trials; 1293 participants; RR 0.88; 95% CI 0.67, 1.16; 95% prediction interval 0.61, 1.27; *I* ^2^ = 0.00; *τ* ^2^ = 0.00) (Table 3).

## 4 Discussion

### 4.1 Main Findings

We found no evidence that antenatal physical activity is associated with improved pregnancy outcomes or reduced gestational weight gain when considering only trials considered to have no or negligible risk of bias. The effect of including trials with increasing potential for bias shifted the estimates of effect further from the null while simultaneously introducing greater heterogeneity.

### 4.2 Interpretation

Our findings are in contrast with those of published systematic reviews and meta‐analyses that have been used to inform national and international guidelines for pregnancy care [1, 2, 3, 4, 7]. In particular, recently published systematic reviews and meta‐analyses have reported that antenatal physical activity interventions are associated with a reduction in gestational diabetes, hypertensive disorders of pregnancy, and gestational weight gain [2, 4, 57, 58]. These aggregate data meta‐analyses included all trials, irrespective of individual trial risk of bias or rigour. We have demonstrated in our review that a small minority of randomised trials have been conducted in a methodologically rigorous way, the majority of trials having substantial risk of bias, particularly in relation to performing an ITT analysis. When considering only trials at no or negligible risk of bias, there is no effect of antenatal physical activity interventions on gestational weight gain or pregnancy and birth outcomes for women and their infants. In contrast, the inclusion of trials with significant methodological flaws results in misleading findings of benefit. Furthermore, almost half of the trials we identified recruited fewer than 100 women. It is well known that smaller studies often show different, commonly larger, treatment effects than those reported by larger randomised trials [59]. Smaller studies, particularly those with fewer than 100 participants, have been shown to inappropriately skew aggregate data meta‐analysis results towards beneficial effects [60]. This has implications for evidence‐based clinical practice guidelines that rely on systematic reviews and meta‐analyses suffering from artificially inflated effect estimates.

The goal of providing evidence‐based healthcare through reliance on evidence‐based clinical practice guidelines is a laudable one. However, this cannot occur when guidelines are based on poor‐quality trials that inappropriately bias aggregate estimates of effect. Additionally, there are potential harms in relying on such biased evidence synthesis. While all pregnancy care guidelines encouraging physical activity state that there is no evidence of harm from such interventions [8, 9, 10, 61, 62], we have shown that the evidence base on which these recommendations rely is of very poor quality, particularly with regard to reporting patient outcomes. Thus it is possible that harms from antenatal physical activity interventions do exist but have not been reported.

It is also worthwhile considering the potential costs of incorporating such misleading systematic review findings into pregnancy care guidelines. Low‐value interventions in healthcare are defined as those that provide little or no clinical benefit, may cause harm, or are not cost‐effective [63]. It has been estimated that up to 25% of national healthcare spending goes towards healthcare waste, including low value interventions [64, 65]. As a result, national and international efforts at deimplementation of ineffective healthcare interventions are underway (Choosing Wisely). A continued focus on low‐value, ineffective interventions such as antenatal physical activity is wasteful and runs the risk of blinding us, as clinicians and researchers, to new and possibly more effective alternatives.

Concerningly, we found only 5 trials to have no or negligible potential for bias due to departures from an ITT analysis, despite some trials reporting that an ITT analysis was performed. A significant proportion of the trials identified in this review excluded women who were not compliant (in either the intervention or control group), rendering these trials fundamentally a per‐protocol analysis. It is well recognised that per‐protocol analyses consistently report greater estimates of treatment effects than ITT analyses, introducing significant bias to aggregated effect estimates [66, 67, 68, 69].

Participants who do not comply with or discontinue a study are likely to not be random in relation to either the intervention or outcome. That is, participants who discontinue the intervention are likely inherently different from participants who continue and adhere, and in turn will be different from control group participants who discontinue a study. This is particularly true of physical activity interventions. In excluding the outcomes of those participants who do not comply with antenatal physical activity interventions, the estimate of intervention effect is particularly prone to bias if no account is taken of informative missing data; in fact it should be considered a different estimand altogether, “analogous to [the comparison arising from] an observational study” [70].

Despite more than half the included trials being published after the introduction of CONSORT reporting guidelines [56], the quality of trial reporting was low. Descriptions of randomisation and allocation concealment were frequently insufficient to adequately assess. While interventions such as antenatal physical activity cannot blind either participant or personnel to allocation, most of the included trials did not provide sufficient information to assess whether outcome assessors were blinded. Loss to follow‐up was generally high, and only a minority of trials undertook statistical analysis taking this into account.

Since 2005, it has been a requirement of the International Committee of Medical Journal Editors (ICMJE) that clinical trials are prospectively registered in a public registry [71]. Additionally, it is a requirement of journal editors that this is enforced [72]. Retrospective trial registration has been associated with a higher risk of bias across all domains of quality assessment, emphasising the importance of this requirement [73]. Unfortunately, our findings are consistent with those of previous reviews investigating compliance with this reporting requirement among obstetric and gynaecological randomised trials [73, 74]. It is clear that improvement in trial quality and reporting is urgently required. Any future studies in this field should be prospectively registered, appropriately powered, and avoid excluding non‐compliant participants. Additionally, investigation of the effects of physical activity interventions that are scalable and affordable on a population level is important. We found significant heterogeneity with regards to the type and intensity of interventions assessed, raising doubt as to whether such disparate trials should be combined in a meta‐analysis.

### 4.3 Strengths and Limitations

A major strength of our work is the rigorous search and interrogation of the included trials. We undertook extensive cross‐checking between trial registrations, published protocols (where present), and trial reports (including history of changes) to define a single primary publication and appropriately define trials as registered retrospectively or prospectively. A further strength is the comprehensive and detailed assessment of the potential for bias, defined as the degree of deviation from an ITT analysis. This unique assessment was undertaken using robust statistical principles and facilitated sequential meta‐analysis to demonstrate the effects of the inclusion of studies with increasingly greater potential for bias on the effect estimates.

## 5 Conclusions

Our findings cast doubt on the validity of national and international clinical practice guidelines recommending antenatal physical activity for improvement of pregnancy outcomes. A rigorous assessment of trial quality is required prior to incorporation of evidence synthesis into such clinical practice guidelines, as we found a significant proportion of included trials were of poor quality and biased aggregate estimates of effect.

## Author Contributions

Each author fulfils the requirements for authorship. All authors, A.J.P., A.R.D., M.M., L.S., J.L., J.M.D. have been involved equally in the concept and design of this review, screening of studies, data extraction and analysis and interpretation, critical revision of the manuscript for important intellectual content, and provide approval of the final submitted version. J.L. was responsible for conducting the statistical analyses. A.J.P. drafted the manuscript, had full access to all of the study data, and takes responsibility for the integrity of the data, and accuracy of the data analysis.

## Conflicts of Interest

Jodie M Dodd is funded through NHMRC Investigator Grant (ID 1196133). All authors contributed to the conception, design, performance, analysis, and writing of this work. The authors have no conflicts of interest to declare.

## Supporting information


**Figure S1.** Search strategy for Ovid MEDLINE database.
**Figure S2**. Effect of intervention and risk of bias on maternal and infant outcomes.

## Data Availability

The data presented in this analysis are available for discussion and review with the authors.
