# Supplementary material for: Antenatal Physical Activity Interventions and Pregnancy Outcomes: A Systematic Review and Meta‐Analysis With a Focus on Trial Quality
Source: BJOG. 2025 Feb 3;132(6):709–23. doi: 10.1111/1471-0528.18084 (PMC11969922; doi:10.1111/1471-0528.18084)
Supplement: Supplementary file 1 — Figure S1. Search strategy for Ovid MEDLINE database. Figure S2. Effect of intervention and risk of bias on maternal and infant outcomes. [file BJO-132-709-s001.zip › BJO18084-sup-0001-Fig S1 search strategy.docx]

**Fig. S1**: Search strategy for Ovid MEDLINE database

1. Pregnant women/
2. Exp Pregnancy/
3. Perinatal care/ or prenatal care/
4. (pregnan* or maternal or gestation* or perinatal or prenatal or antenatal).ti,ab.
5. Preconception care/
6. Exp Obesity, maternal/
7. Female/
8. Overweight/
9. 7 AND 8
10. Maternal.mp.
11. Gestation.mp.
12. Gravid.mp.
13. Prenatal care.mp
14. Exp Pregnancy complications/
15. 1 AND 8
16. Or/1-5, 9-15
17. Body weight
18. Exp body weight changes/
19. Body mass index/
20. (weight or overweight or obes* or BMI).ti,ab
21. Exp Gestational weight gain/
22. OR/17-21
23. AND/16, 22
24. Exp life style/
25. Exp diet therapy/
26. Exp exercise/
27. Exp physical fitness/
28. Counseling or
29. exp directive counseling/
30. Exp behaviour therapy/
31. Exp motivation/
32. *Health behaviour/
33. Risk reduction behaviour/
34. Patient education as topic/
35. Health education/
36. Exp health promotion/
37. Diet/
38. Prenatal education/
39. OR/24-38
40. AND/23, 39
41. Randomised controlled trials as topic/
42. Clinical trial/
43. Controlled clinical trial/
44. Randomised trial.ti,ab.
45. Exp clinical trial/
46. Exp controlled clinical trial/
47. Clinical trial.ti,ab
48. (random* or control* or cohort or trial).ti,ab,kw.
49. Limit 40 to humans
50. OR/41-48
51. 49 AND 50
