# Supplementary material for: Antenatal Physical Activity Interventions and Pregnancy Outcomes: A Systematic Review and Meta‐Analysis With a Focus on Trial Quality
Source: BJOG. 2025 Feb 3;132(6):709–23. doi: 10.1111/1471-0528.18084 (PMC11969922; doi:10.1111/1471-0528.18084)

Supplementary Figure 2. Effect of intervention and risk of bias on maternal and infant outcomes

a) Infant birthweight

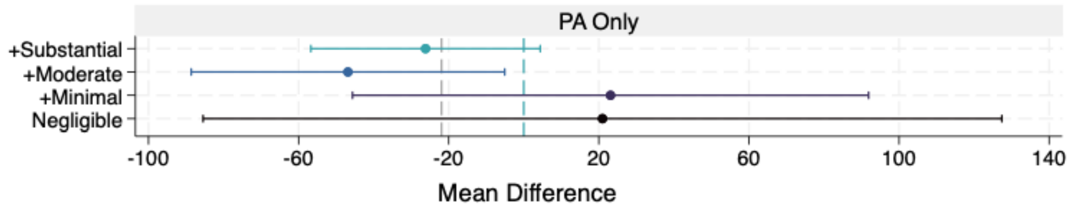

b) Gestational diabetes mellitus

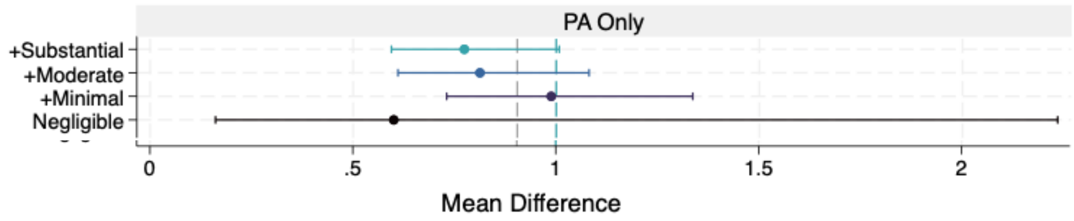

c) Preeclampsia

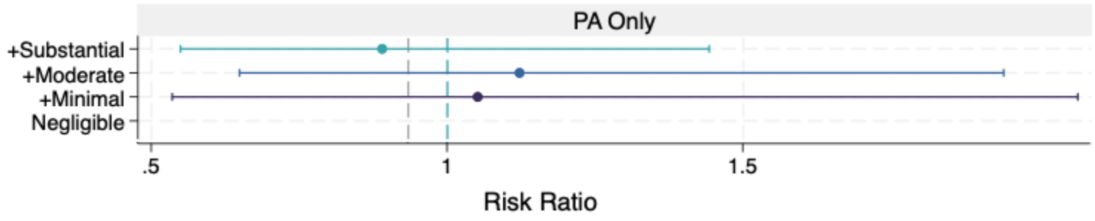

d) LSCS

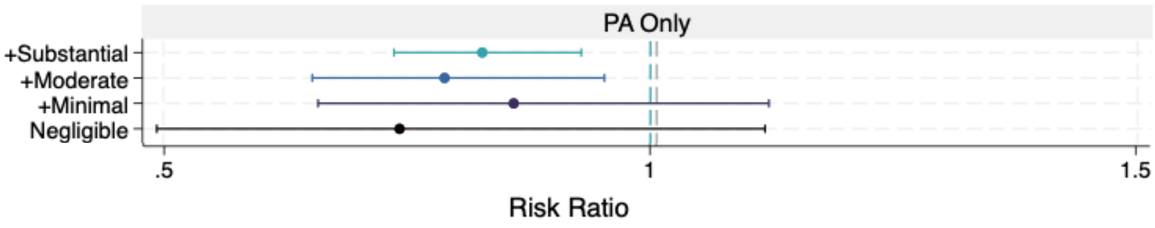

e) Gestational weight gain

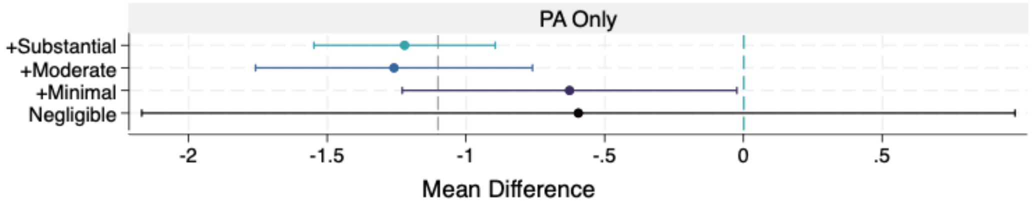

f) Infant large for gestational age

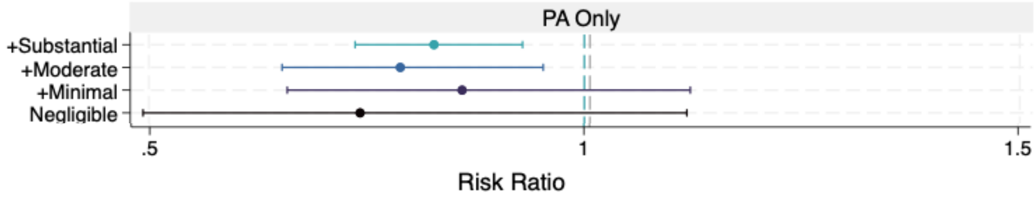

Supplement: Supplementary file 1 — Figure S1. Search strategy for Ovid MEDLINE database. Figure S2. Effect of intervention and risk of bias on maternal and infant outcomes. [file BJO-132-709-s001.zip › BJO18084-sup-0003-Supplementary Figure 2.pdf]
