# Supplementary material for: Antenatal Physical Activity Interventions and Pregnancy Outcomes: A Systematic Review and Meta‐Analysis With a Focus on Trial Quality
Source: BJOG. 2025 Feb 3;132(6):709–23. doi: 10.1111/1471-0528.18084 (PMC11969922; doi:10.1111/1471-0528.18084)

**Fig. S1**: Search strategy for Ovid MEDLINE database

1. Pregnant women/
2. Exp Pregnancy/
3. Perinatal care/ or prenatal care/
4. (pregnan* or maternal or gestation* or perinatal or prenatal or antenatal).ti,ab.
5. Preconception care/
6. Exp Obesity, maternal/
7. Female/
8. Overweight/
9. 7 AND 8
10. Maternal.mp.
11. Gestation.mp.
12. Gravid.mp.
13. Prenatal care.mp
14. Exp Pregnancy complications/
15. 1 AND 8
16. Or/1-5, 9-15
17. Body weight
18. Exp body weight changes/
19. Body mass index/
20. (weight or overweight or obes* or BMI).ti,ab
21. Exp Gestational weight gain/
22. OR/17-21
23. AND/16, 22
24. Exp life style/
25. Exp diet therapy/
26. Exp exercise/
27. Exp physical fitness/
28. Counseling or
29. exp directive counseling/
30. Exp behaviour therapy/
31. Exp motivation/
32. *Health behaviour/
33. Risk reduction behaviour/
34. Patient education as topic/
35. Health education/
36. Exp health promotion/
37. Diet/
38. Prenatal education/
39. OR/24-38
40. AND/23, 39
41. Randomised controlled trials as topic/
42. Clinical trial/
43. Controlled clinical trial/
44. Randomised trial.ti,ab.
45. Exp clinical trial/
46. Exp controlled clinical trial/
47. Clinical trial.ti,ab
48. (random* or control* or cohort or trial).ti,ab,kw.
49. Limit 40 to humans
50. OR/41-48
51. 49 AND 50

**Supplementary Figure 2.** Effect of intervention and risk of bias on maternal and infant outcomes

1.
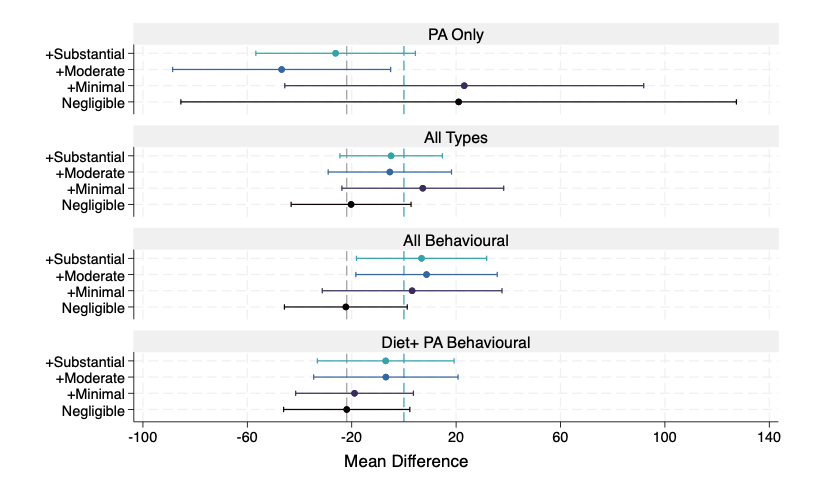

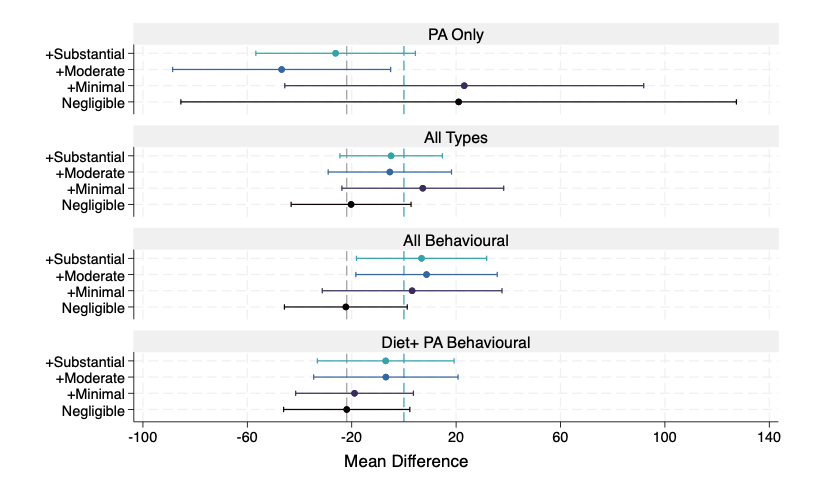
Infant birthweight
2. Gestational diabetes mellitus
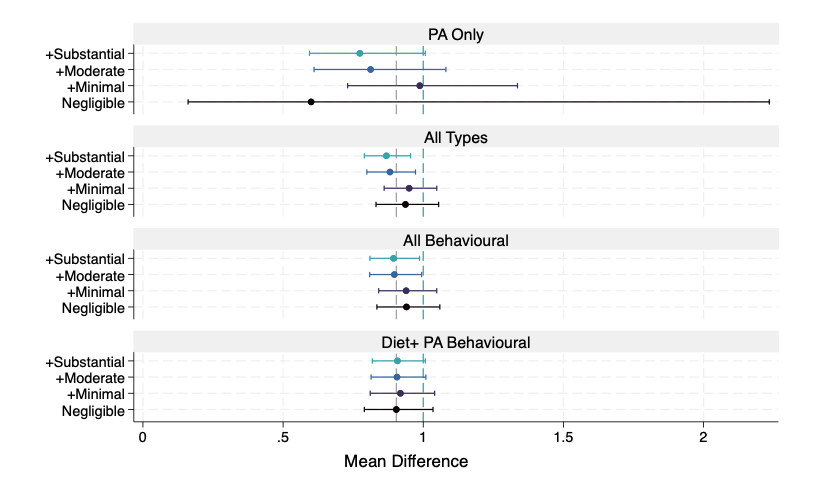

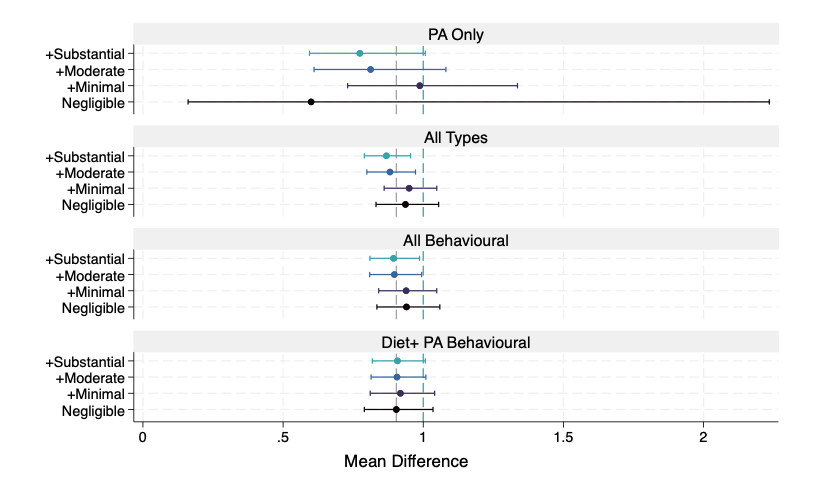

3.
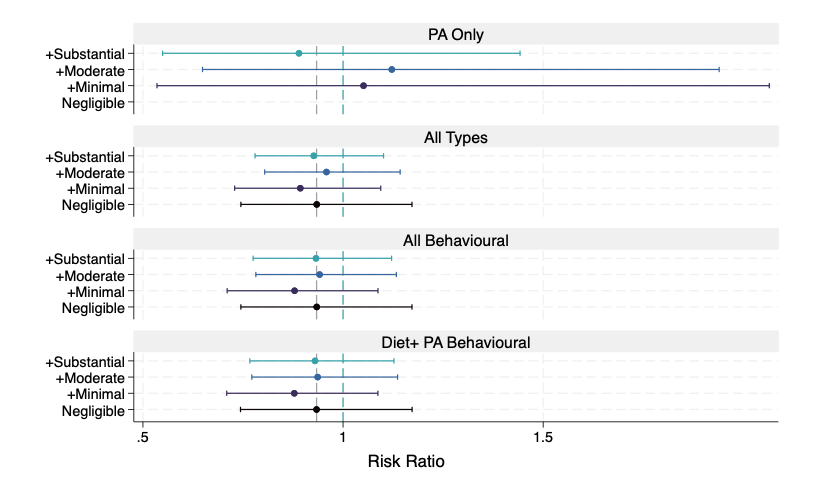

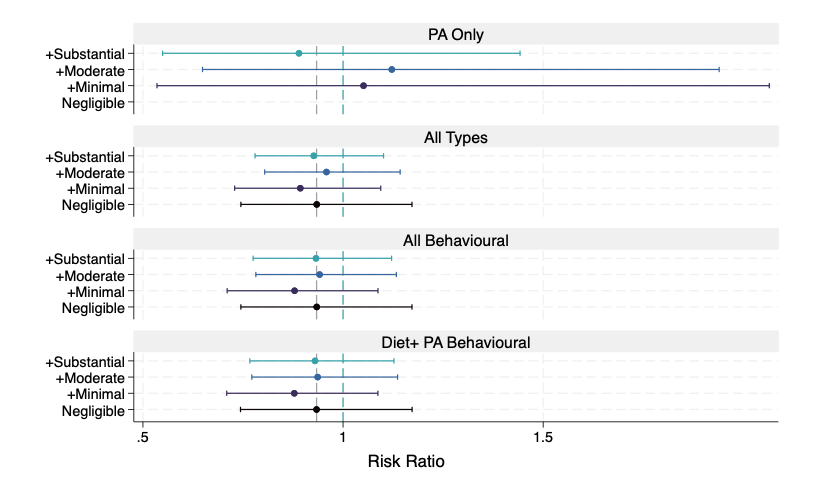
Preeclampsia
4.
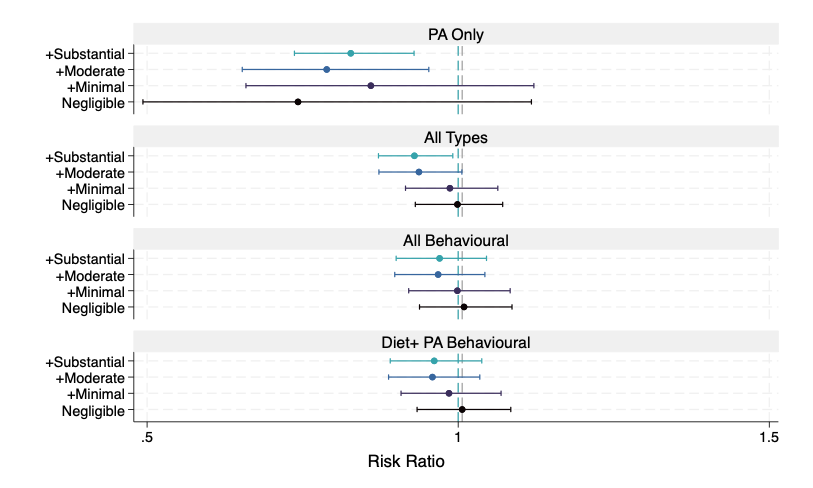

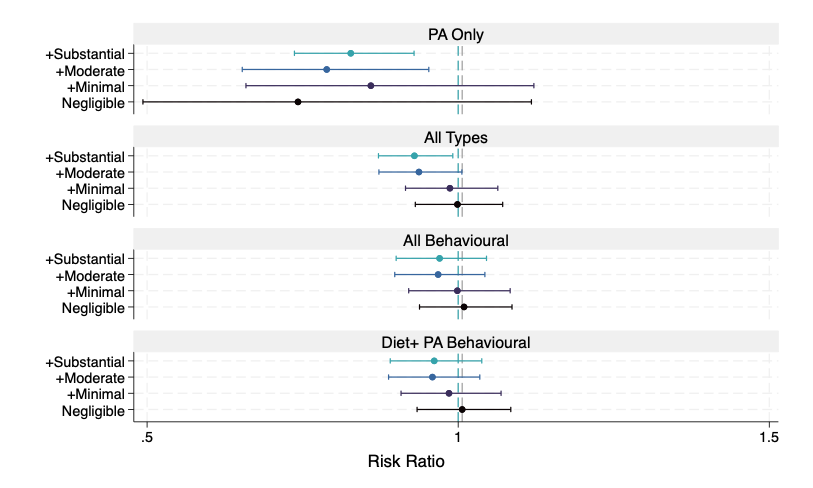
LSCS
5. Gestational weight gain


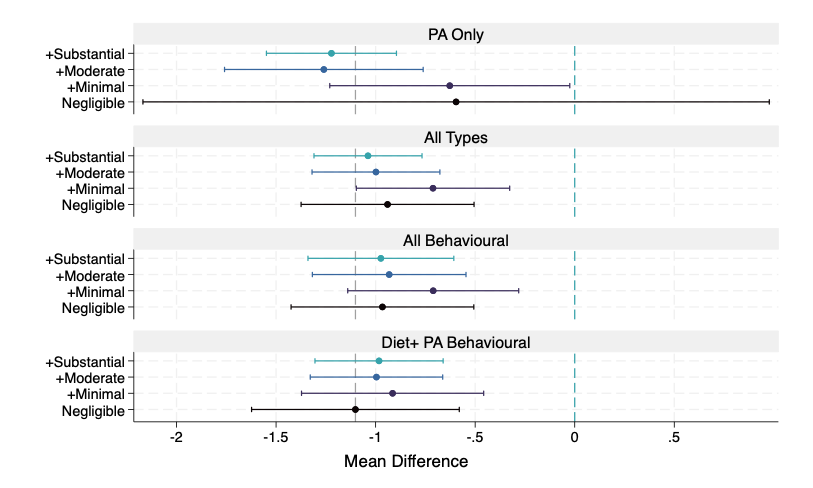


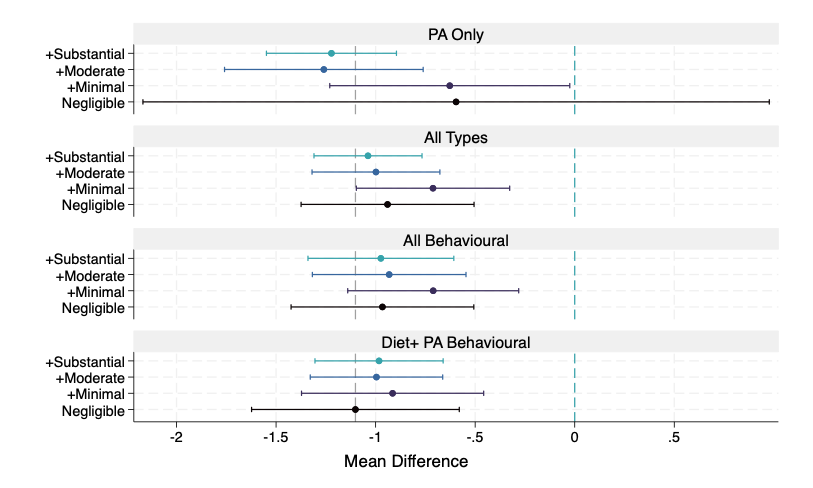


1. Infant large for gestational age


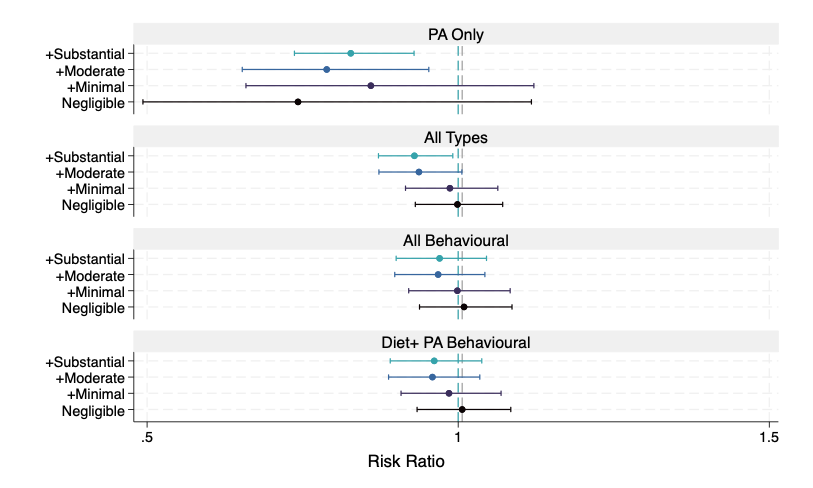

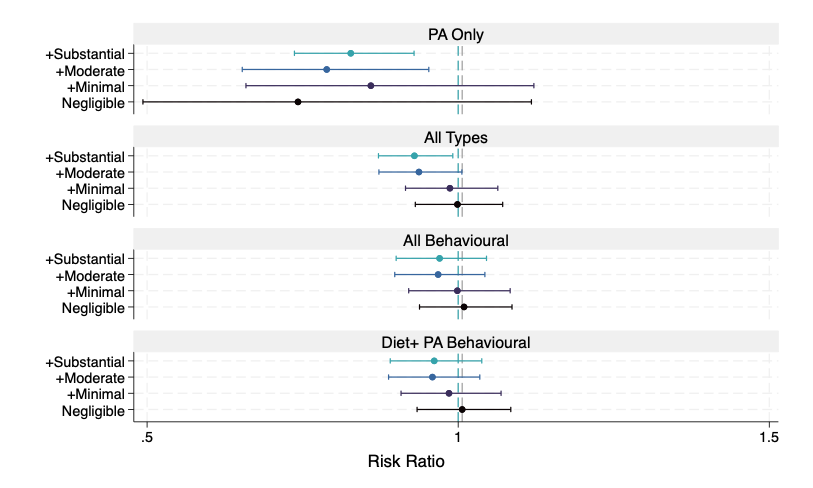

Supplement: Supplementary file 1 — Figure S1. Search strategy for Ovid MEDLINE database. Figure S2. Effect of intervention and risk of bias on maternal and infant outcomes. [file BJO-132-709-s001.zip › Fig S1-S2.docx]
